# Large-Scale Transcriptome Analysis of Cucumber and *Botrytis cinerea* during Infection

**DOI:** 10.1371/journal.pone.0142221

**Published:** 2015-11-04

**Authors:** Weiwen Kong, Nan Chen, Tingting Liu, Jing Zhu, Jingqi Wang, Xiaoqing He, Yi Jin

**Affiliations:** College of Biological Sciences and Technology, P. O. Box 162, Beijing Forestry University, Beijing 100083, China; Seoul National University, REPUBLIC OF KOREA

## Abstract

Cucumber gray mold caused by *Botrytis cinerea* is considered one of the most serious cucumber diseases. With the advent of Hi-seq technology, it is possible to study the plant–pathogen interaction at the transcriptome level. To the best of our knowledge, this is the first application of RNA-seq to identify cucumber and *B*. *cinerea* differentially expressed genes (DEGs) before and after the plant–pathogen interaction. In total, 248,908,688 raw reads were generated; after removing low-quality reads and those containing adapter and poly-N, 238,341,648 clean reads remained to map the reference genome. There were 3,512 cucumber DEGs and 1,735 *B*. *cinerea* DEGs. GO enrichment and KEGG enrichment analysis were performed on these DEGs to study the interaction between cucumber and *B*. *cinerea*. To verify the reliability and accuracy of our transcriptome data, 5 cucumber DEGs and 5 *B*. *cinerea* DEGs were chosen for RT-PCR verification. This is the first systematic transcriptome analysis of components related to the *B*. *cinerea*–cucumber interaction. Functional genes and putative pathways identified herein will increase our understanding of the mechanism of the pathogen–host interaction.

## Introduction

Cucumber (*Cucumis sativa* Linn) belongs to the Cucurbitaceous family and is one of the world’s most economically valuable and nutritional vegetable crops. It has been widely cultivated worldwide, especially in Southeast Asia [1]. Cucumber is highly susceptible to necrotrophic fungal pathogens, *B*.*cinerea* being the most common [2]. Cucumber gray mold is considered one of the most serious cucumber diseases and is caused by *B*. *cinerea* [3]. Besides cucumber, it can also infect more than 200 types of plants [4, 5], resulting in serious economic losses.

Many studies have explored necrotrophic plant pathogens during the past few decades. *B*.*cinerea* is one of the most comprehensively studied necrotrophic fungal plant pathogens [6] and can attack several plant tissues such as stems, flowers, and leaves; establish a necrotrophic interaction; and kill host cells to obtain nutrition [7]. Pathogenic bacteria can proliferate in intercellular spaces after entering plant organs through gas or water pores, and if the plant has wounds, they can also invade the inside of the plants [8]. *B*.*cinerea* has evolved complex strategies to evade the plants’ immune system [9]. For example, when *B*. *cinerea* infects the host, it can destroy the cell walls by secreting diverse proteins and enzymes [6, 10]. Polygalacturonase BcPG1 is known to be an important virulence factor during infection [11, 12]. The expression patterns of the encoding genes (Bcpg 1–6) have been studied on four hosts: tomato, broad bean, apple, and courgette [13, 14].

Throughout their life cycle, plants are exposed to various threats from the outside environment. Plants have also evolved complex strategies, collectively called “defense” or “tolerance” responses, to protect themselves against unstable environmental conditions such as drought, low temperature, salinity, and infections by pathogenic microbes [15–17]. Pathogen infection is one of the most serious dangers, which plants must cope with. Unlike animals, plants do not have mobile defender cells and adaptive immune systems comprising somatic cells. However, each cell has innate immunity and can recognize systemic signals originating from infection sites [11]. The mechanisms of plant resistance have been studied in recent years. Reactive oxygen species (ROS) are the ubiquitous early component of the resistance mechanism of plant cells in response to pathogen attack [18]. Salicylic acid, jasmonic acid, and ethylene (ET) are three phytohormones that play important roles in the regulation of transduction pathways and can defend against pathogen infection. Among these phytohormones, jasmonic acid and ET play major roles in the activation of defense responses against necrotrophic pathogen attack [13, 15, 19, 20]. In addition, hypersensitive reactions can improve plant defense against pathogens to *Arabidopsis* spp. Cell death can be a programmed event that occurs when plants are attacked by pathogens [21].

Mechanisms of the host—pathogen interaction have always been important issues. Recent studies have focused on the physiology and biochemistry of several specific gene expression changes after pathogen invasion. With the advent of RNA-seq technologies, it is possible for us to observe whole transcriptome changes. By comparing these transcriptome changes, we can identify host-related or pathogen-related genes and determine which metabolic pathways function during pathogen invasion. To date, studies on host transcriptome changes after inoculation with *B*. *cinerea* have only been reported in *Arabidopsis thaliana*, tomato and *Lactuca sativa* [7], and only changes in the plant transcriptome have been investigated.

In the present study, we examined the genome-wide gene expression profile of *B*. *cinerea* and cucumber before and after infection using Illumina sequencing and bioinformatics analysis. After RNA-seq, we used recently sequenced cucumber and *B*. *cinerea* genomes as reference genomes. RT-PCR was also applied to validate the results of RNA-seq. The primary objective of this study was to annotate functional genes from this transcriptome analysis and identify genes and pathways involved in the plant—pathogen interaction. To the best of our knowledge, this is the first analysis of large-scale transcriptome changes during the cucumber–*B*. *cinerea* interaction. These results will increase our understanding of the molecular mechanisms of the cucumber–*B*. *cinerea* interaction and may be used to protect plants against disasters caused by necrotrophic fungal pathogens.

## Materials and Methods

### Biological materials

*B*.*cinerea* strain B05.10 (provided by China General Microbiological Culture Collection Center) was cultured, and spores were harvested according to Broekaert et al. [22]. Seeds of *C*. *sativus* L. were obtained from the Institute of Vegetables and Flowers, Chinese Academy of Agriculture Science.

### Plant growth and inoculation

*Cucumis sativus* L. seeds were cultivated for 4 weeks (four leaves) in MS culture at 22°C under a 12 h daylight cycle. Two 5 μL drops of a *B*. *cinerea* spore suspension (5 × 10^5^ mL^-1^ in 1/2 PDB) were inoculated onto every leaf per plant. Mock-inoculated leaves received only 1/2 PDB [13]. Pure culture of *B*. *cinerea* was used as a control. At 96 h after inoculation, we moved the plant samples and pure culture of *B*. *cinerea* into liquid nitrogen and stored the samples at –80°C for RNA extraction.

### RNA isolation, cDNA library construction, and sequencing

Parallel massive sequencing of cDNA (RNA-seq) was used to measure the genome-wide expression levels of genes from the fungus and host, which were cultured under isolated conditions. Next, the fungus was inoculated into the host. When the host was fully infected, the same sets of transcriptional genes were measured using deep sequencing. Because of infection and anti-infection, some genes from the pathogen and host alter their regulation to maximize resource utilization efficiency. RNA purity was checked using the Nano Photometer^®^ spectrophotometer (IMPLEN, CA, USA). The RNA concentration was measured using the Qubit^®^ 2.0 Flurometer (Life Technologies, CA, USA). RNA integrity was assessed using the RNA Nano 6000 Assay Kit of the Bioanalyzer 2100 system (Agilent Technologies, CA, USA). cDNA library construction and Illumina deep sequencing were performed following the method of Li [23], and 100-bp paired-end reads were generated.

### Quality control

Raw data (raw reads) of fastq format were first processed through in-house perl scripts. In this step, clean data (clean reads) were obtained by removing low-quality reads and those containing adapter and poly-N from the raw data. At the same time, the Q20, Q30, and GC content of the clean data were calculated. All downstream analyses were based on the clean data of high quality.

### Read mapping to the reference genome

*Cucumis sativus* L. and *B*. *cinerea* reference genomes and their corresponding gene model annotation files were downloaded from ftp://www.icugi.org/pub/genome/cucumber/ and ftp://ftp.ensemblgenomes.org/pub/release-23/fungi/fasta/botryotinia_fuckeliana/dna/ Indexes of the reference genomes were built using Bowtie v2.0.6, and paired-end clean reads were aligned to the reference genome using TopHat v2.0.9.

### Quantification of gene expression level and differential expression analysis

HTSeq v0.6.1 was used to count the read numbers mapped to each gene [24]. The RPKM of each gene was calculated based on the length of the gene and read counts mapped to this gene. RPKM, Reads Per Kilobase of exon model per Million mapped reads, considers the effect of sequencing depth and gene length for the reads count at the same time, and is currently the most commonly used method for estimating gene expression levels [25]. Prior to differential gene expression analysis, for each sequenced library the read counts were adjusted using the edgeR program package through one scaling normalized factor. Differential expression analysis of two conditions was performed using the DEGSeq R package (1.12.0). A corrected P-value of 0.005 and log2 (fold change) of 1 were set as the threshold for significantly differential expression.

### Gene Ontology and KEGG enrichment analysis of differentially expressed genes

Gene Ontology (GO) enrichment analysis of differentially expressed genes was implemented using the GOseq R package, in which gene length bias was corrected. GOseq method is based on Wallenius non-central hyper-geometric distribution, compared with ordinary hyper-geometric distribution, this method could calculate the probability of GO term enriched by differencial expression genes more accurately.[26]. GO terms with corrected P values of <0.05 were considered significantly enriched by DEGs. KOBAS software was used to examine the statistical enrichment of DEGS in The Kyoto Encyclopedia of Genes and Genomes (KEGG) pathways [27, 28].

### Gene expression validation

To validate the DEGs data obtained by RNA sequencing in cucumber and *B*. *cinerea*, qRT-PCR was performed on five cucumber DEGs and five *B*. *cinerea* DEGs with total RNA used for RNA-seq (Table 1). Primers were designed according to Illumina sequencing data with Primer Premier 5 (S1 Table). A Quant One Step RT-PCR kit (Tiangen, Beijing) was used to prepare cDNA from isolated RNA. Real-time quantification was performed in triplicate with FastFire qPCR PreMix SYBR Green (Tiangen, Beijing) in MX3005P, Stratagene, using the following program: 95°C for 10 min followed by 45 cycles of 95°C for 10 s and 60°C for 1 min. The levels of DEGs were normalized against the cucumber *actin3* gene and *B*. *cinerea* actin gene, respectively.

**Table 1:** Genes used for qRT- PCR analyses.

Gene ID	Description	log2fold change	p-value	q-value
Csa5G165850	ethylene-responsive transcription factor 2	2.4777	1.37E-14	1.70E-13
Csa3G198490	Probable indole-3-acetic acid-amido synthetase GH3.1	4.2956	8.42E-73	5.77E-71
Csa1G600830	Abscisic Acid-Insensitivate 5-like protein 2	2.342	1.41E-06	8.37E-06
Csa1G008570	hypersensitive-induced response protein 1	1.8701	7.13E-20	1.23E-18
Csa1G534750	endochitinase PR4-like	4.0896	1.17E-35	3.77E-34
B0510_2072	Botryotinia fuckeliana endopolygalacturonase 4	4.2206	2.48E-26	2.89E-25
B0510_8612	similar to chitinase A	-1.81	9.28E-11	4.98E-10
B0510_8082	hypothetical protein	-1.9088	4.53E-05	0.000132
B0510_958	isocitrate dehydrogenase subunit 1, mitochondrial precursor	1.6458	9.33E-36	1.47E-34
B0510_903	hypothetical protein	-1.1253	2.84E-06	9.79E-06

## Results

### Transcriptome sequencing and mapping to the reference genome

In total, 248,908,688 raw reads were generated; after removing low-quality reads and those containing adapter and poly-N, 238,341,648 clean reads remained (Table 2). The remaining clean data were used to map to the reference genome. In the plant, control samples had 90.29% of reads mapped to the genome and treated samples had 84.87% mapped to the genome. In *B*. *cinerea*, the percents of controlled and treated samples were 84.21% and 3.92%, respectively.

**Table 2:** Summary of sequences analysis.

Sample	Raw reads	Clean reads	Clean bases	Error (%)	Q20 (%)	Q30 (%)	GC (%)
CSV_1	34594807	33137628	4.14G	0.03	96.97	93.76	44.08
CSV_2	34594807	33137628	4.14G	0.04	94.14	89.24	43.97
BC_1	29169817	27961104	3.5G	0.03	97.02	93.75	46.76
BC_2	29169817	27961104	3.5G	0.04	93.91	88.77	46.65
CSVBC_1	60689720	58072092	7.26G	0.03	97.17	94.30	44.11
CSVBC_2	60689720	58072092	7.26G	0.03	95.35	91.33	44.08
Summary	248908688	238341648	29.8G				

CSV: Control Cucumber

BC: Control *B*. *cinerea*

CSVBC: Cucumber infected with *B*. *cinerea*

CSV_1: Reads sequencing of control root from the left.

CSV_2: Reads sequencing of control root from the right.

Q20: The percentage of bases with a Phred value >20.

Q30: The percentage of bases with a Phred value >30.

### Differential gene expression between control and treated samples

**Fig 1:**
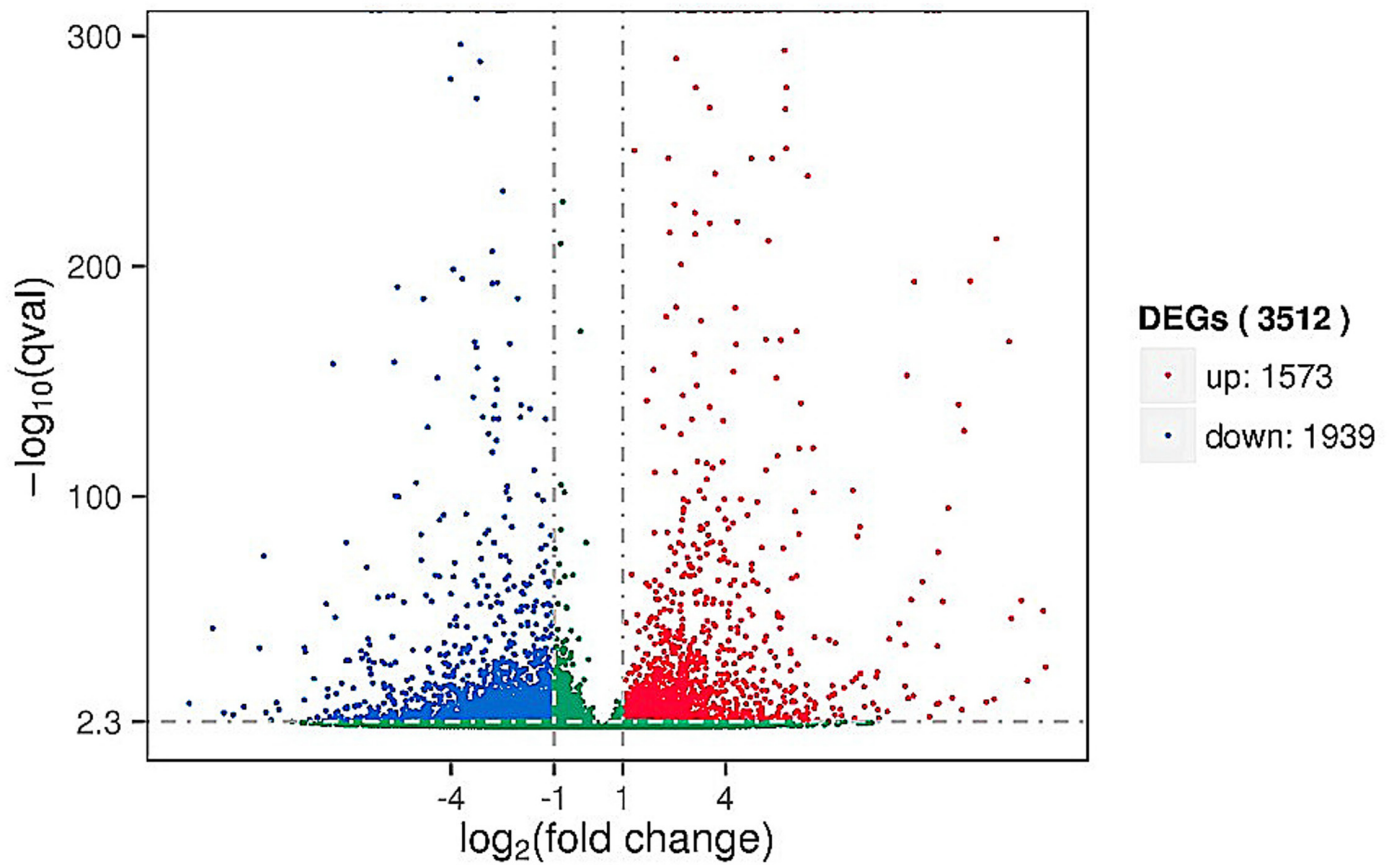
Volcano map of differentially expressed cucumber genes. Significantly differentially expressed genes are shown as a red (up) or green (down) dot. No significant difference between the expressions of genes is shown as a blue dot. Abscissa represents multiple genes expressed in different samples. Ordinate represents the magnitude of gene expression changes.

**Fig 2:**
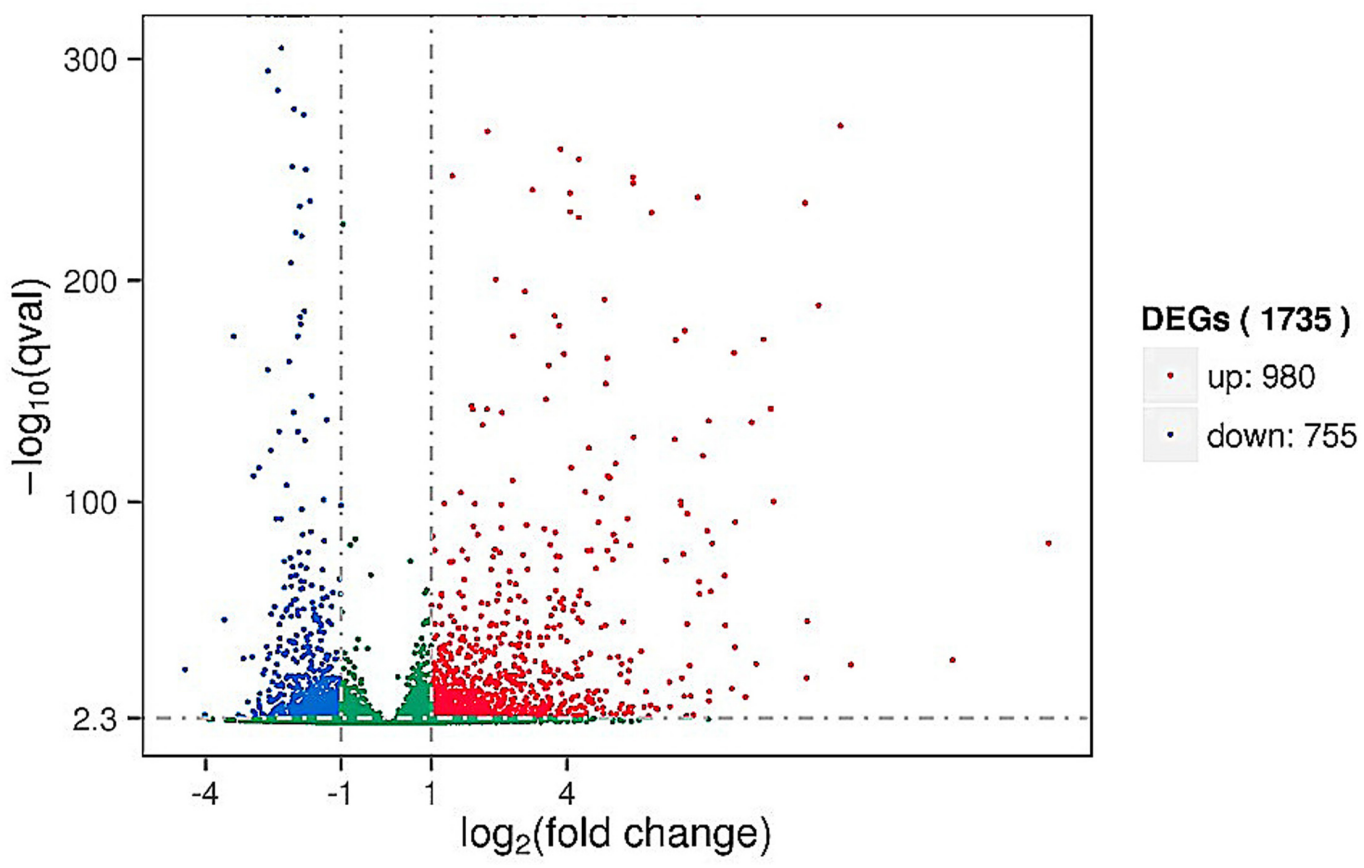
Volcano map of differentially expressed *B*. *cinerea* genes. Significantly differentially expressed genes are shown as a red (up) or green (down) dot. No significant difference between the expressions of genes is shown as a blue dot. Abscissa represents multiple genes expressed in different samples. Ordinate represents the magnitude of gene expression changes.

The expected number of reads per kilobase of transcript sequence per million base pairs sequenced (RPKM) is the most common method with which to estimate the level of gene expression [25]. TMM was used to standardize the read count data obtained from the last step, and DEGSeq was used to examine the differential gene expression profile between the control and treated samples. There was a total of 3,512 DEGs in the plants; 1,753 DEGs were upregulated and 1,939 were downregulated (Fig 1). There was a total of 1,735 DEGs in the pathogen; 980 DEGs were upregulated and 755 were downregulated (Fig 2). Detailed DEGs for both *C*. *sativus* L. and *B*. *cinerea* are provided in S2 and S3 Tables.

### GO enrichment of DEGs for the host and pathogen

GO (http://www.geneontology.org/) is an international standard classification system for gene function, including biological processes, cellular components, and molecular function. GO enrichment analysis of cucumber and *B*. *cinerea* was conducted to characterize the DEG profiles discussed above. GO enrichment results of all DEGs are provided in S4 and S5 Tables.

**Fig 3:**
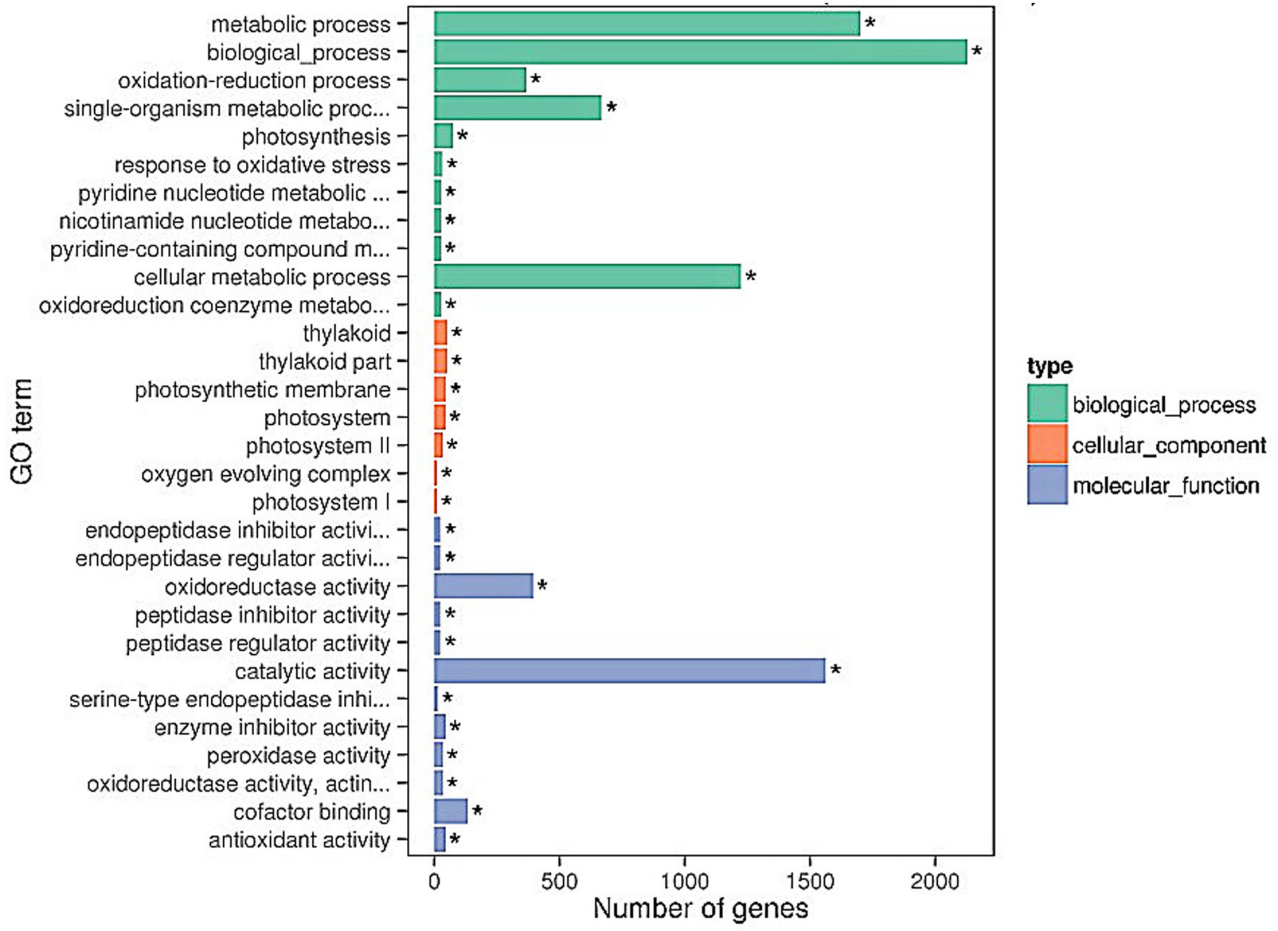
The 30 most enriched GO terms (cucumber). Bar chart of cucumber DEGs enriched in GO term, it can directly reflect the number of DEGs distributing into different GO terms.

In the plant (cucumber), a large proportion of cucumber DEGs enriched into the category of biological process were “cellular metabolic process”, “metabolic process”, “oxidation-reduction process”, and “single-organism metabolic process.” In the molecular function category, “catalytic activity”, “oxidoreductase activity”, and “cofactor binding” showed significant proportions. Of genes categorized as cellular components, “oxygen evolving complex photosystem”, “photosynthetic membrane”, and “thylakoid part” were the most enriched terms (Fig 3). There were also many defense responses GO term had been confirmed but involved few DEGs, including “responses to chitin” and “oxidative stress”.

**Fig 4:**
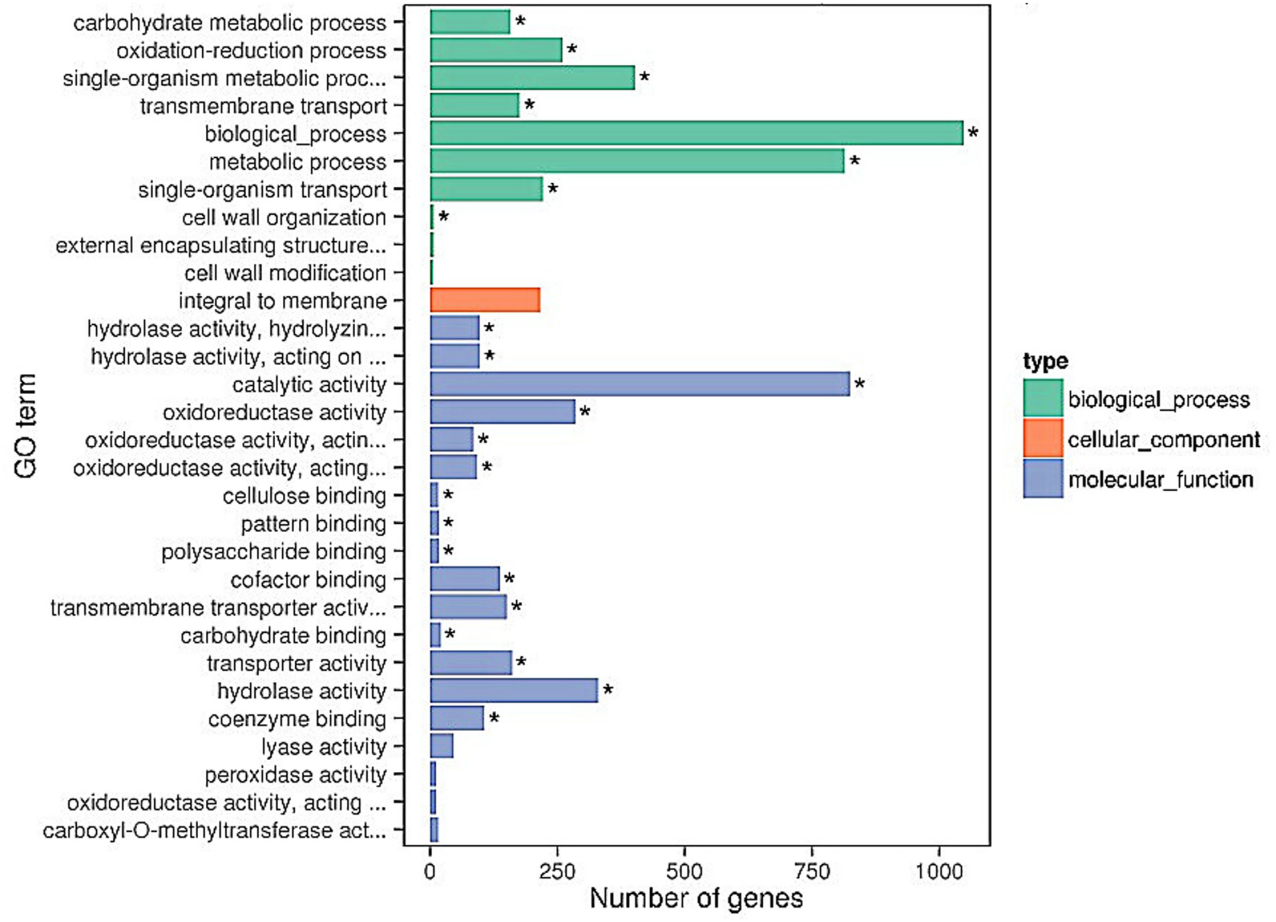
The 30 most enriched GO terms (*B*. *cinerea*). Bar chart of *B*.*cinerea* DEGs enriched in GO term, it can directly reflect the number of DEGs distributing into different GO terms.

In the category of biological process of the pathogen, most DEGs were enriched in “single-organism transport”, “metabolic process”, “transmembrane transport”, “single-organism metabolic process”, and “oxidation-reduction process.” “Integral to membrane” was the dominant subcategory of the cellular component category. In the molecular function category, “catalytic activity”, “oxidoreductase activity”, and “oxidoreductase activity” have shown significant proportions (Fig 4). We also observed genes corresponding to “cell wall modification” GO terms, which had been investigated previously.

### KEGG analysis of plant and pathogen

KEGG is a systematic analysis of gene function and a genome information database. KEGG enrichment analysis of cucumber and *B*. *cinerea* was conducted to identify pathways that play important roles in the plant—pathogen interaction. KEGG enrichment results of all DEGs are shown in S6 and S7 Tables.

**Fig 5:**
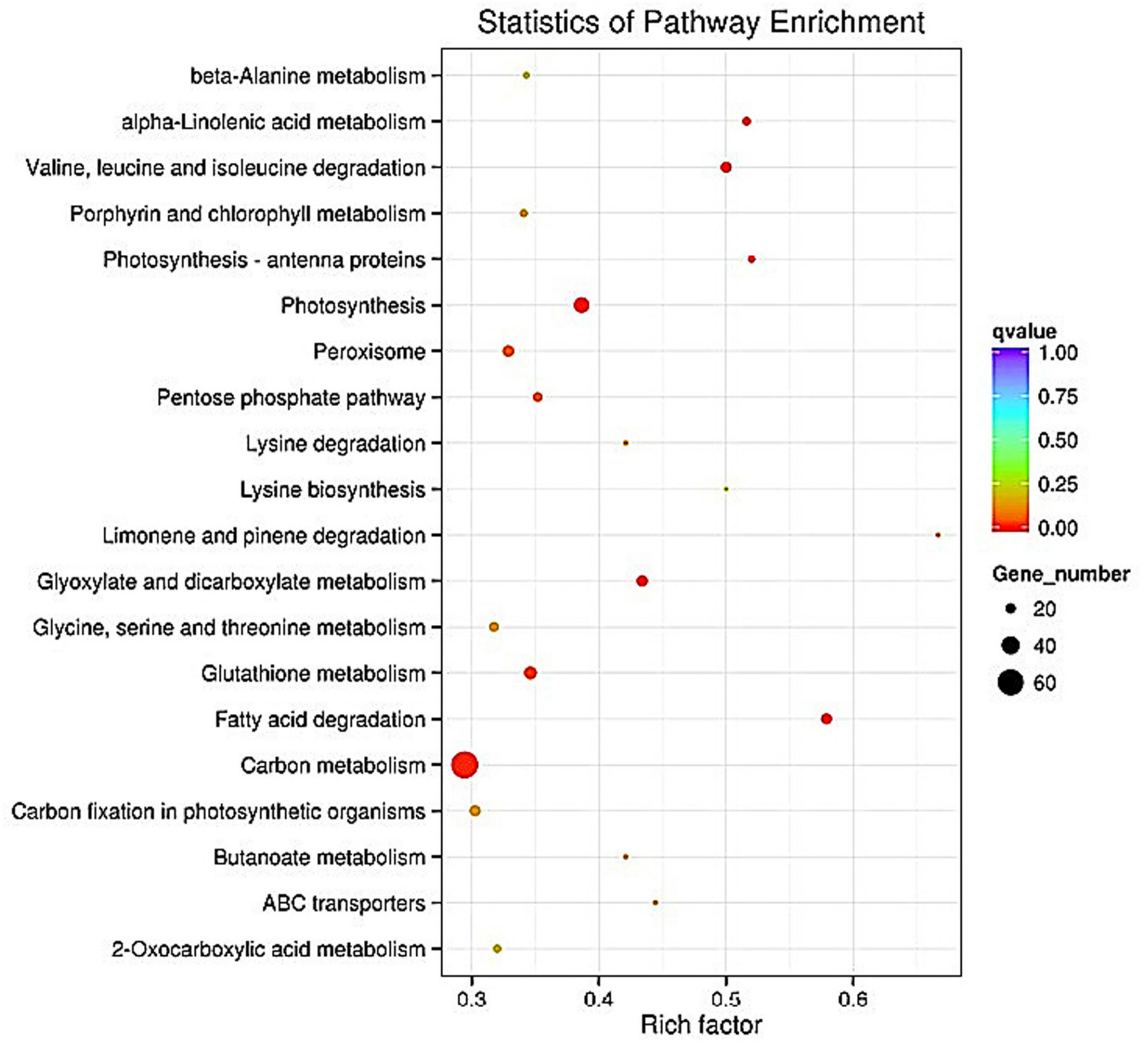
The 20 most enriched KEGG pathways (cucumber). “Rich factor” means that the ratio of the DEGs number and the number of genes have been annotated in this pathway. The greater of the Rich factor, the greater the degree of enrichment.

In the plant, “fatty acid degradation”, “valine, leucine, and isoleucine degradation”, “photosynthesis”, “alpha-linolenic acid metabolism”, and “glyoxylate and dicarboxylate metabolism” were the top five most enriched pathways (Fig 5).

**Fig 6:**
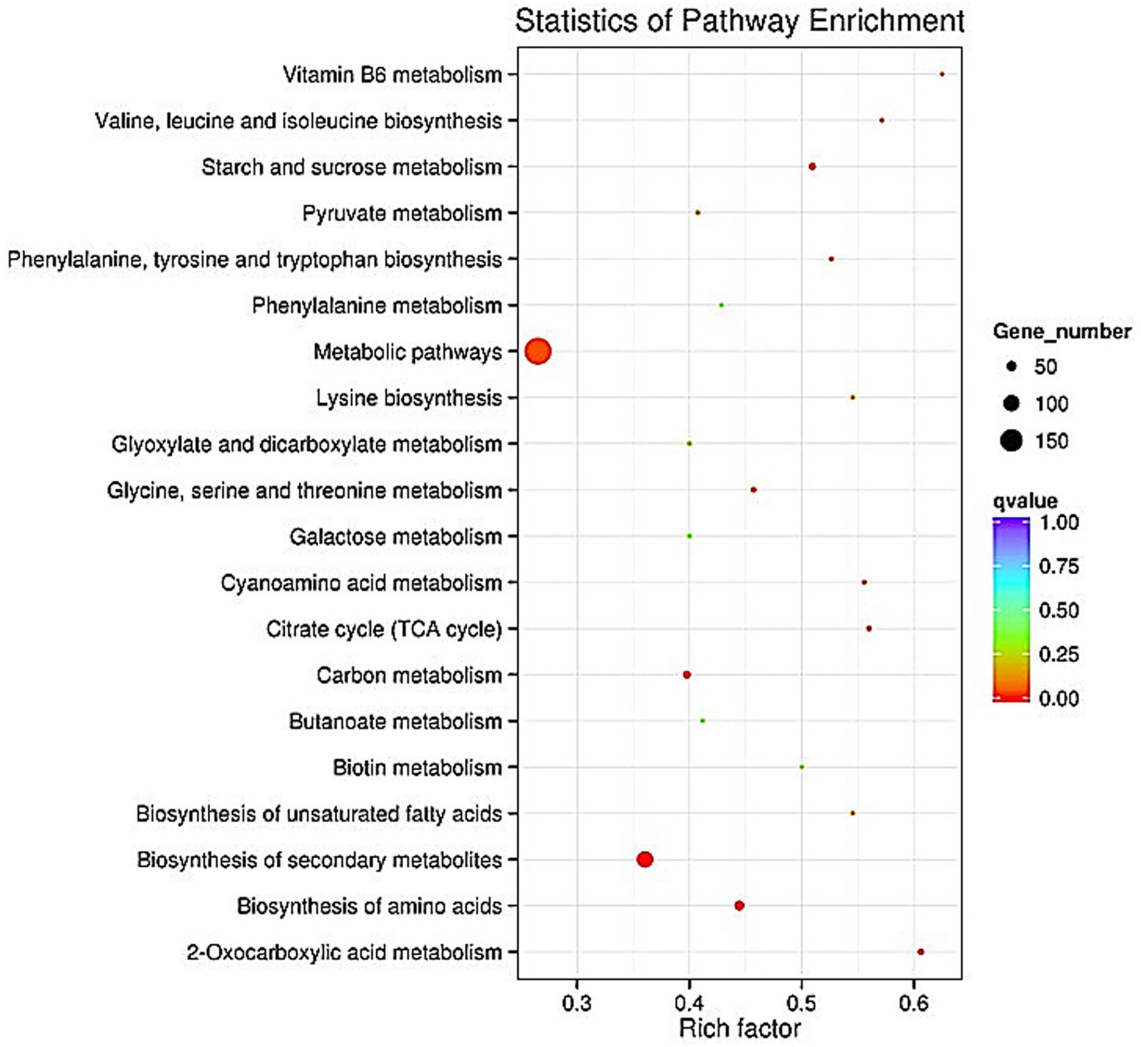
The 20 most enriched KEGG pathways (*B*. *cinerea*). “Rich factor” means that the ratio of the DEGs number and the number of genes have been annoted in this pathway. The greater of the Rich factor, the greater the degree of enrichment.

In the pathogen (*B*.*cinerea*), “biosynthesis of secondary metabolites”, “2-oxocarboxylic acid metabolism”, and “starch and sucrose biosynthesis of amino acid metabolism” were the top three most enriched pathways (Fig 6).

### Real-time quantitative PCR (RT-qPCR) verification

**Fig 7:**
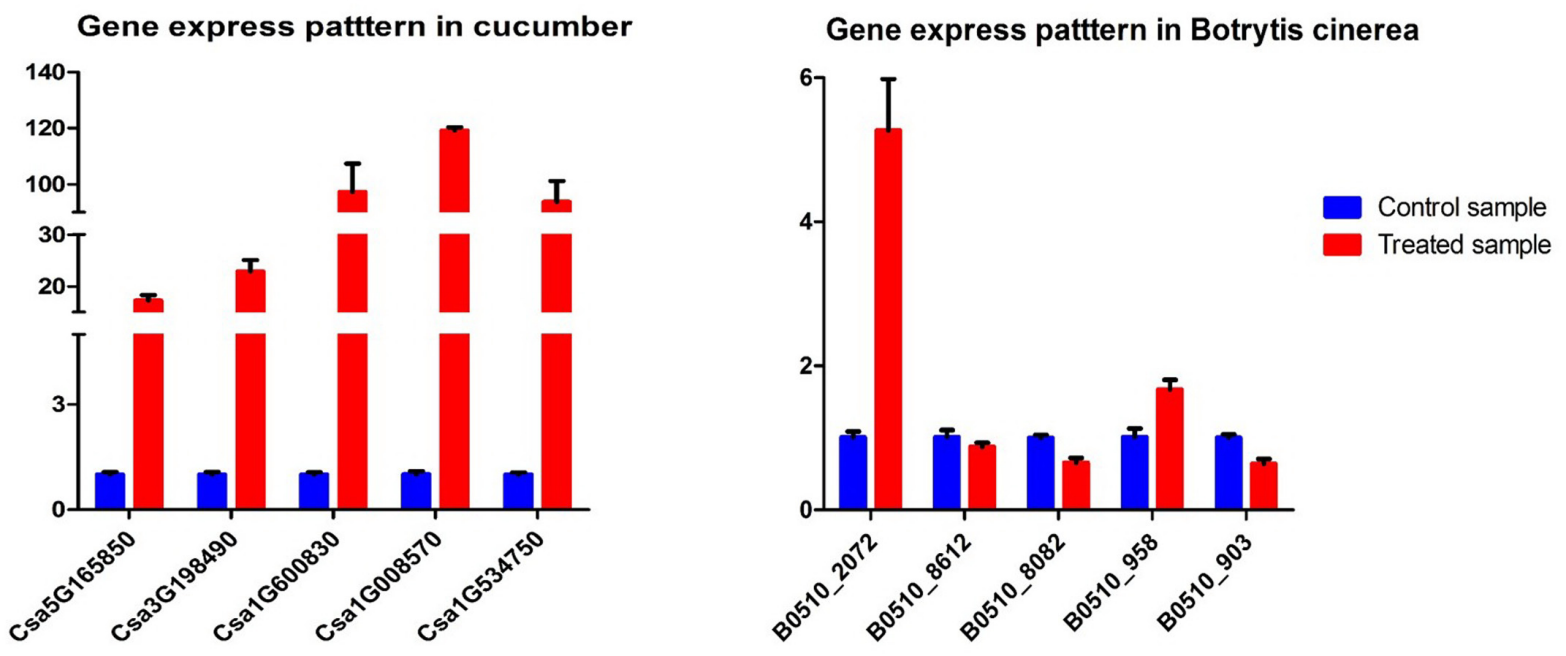
Quantitative RT-PCR validations of five genes in cucumber and five genes in *B*. *cinerea*.

The results of RT-qPCR are shown in Fig 7. Five cucumber DEGs and five *B*. *cinerea* DEGs were selected for RT-qPCR. All genes showed consistent expression patterns with the RNA-Seq data, confirming that our experimental results were valid.

## Discussion

In this study, we investigated whole transcriptome profile changes of cucumber and *B*.*cinerea* before and after infection using RNA-seq. In total, we identified 3,512 cucumber DEGs and 1,735 *B*.*cinerea* DEGs. After enriching these genes into different GO terms and KEGG pathways, we investigated which genes or pathways play important roles in the plant—pathogen interaction. Compared with traditional cDNA microarray technologies, RNA-seq has many advantages: easily detecting low-abundance genes [29], reducing both the cost-per-reaction and time required by orders of magnitude, making use of sequencing as a cost-effective option for many experimental approaches, and higher accuracy and reproducibility [30].

Previous studies examined *Arabidopsis* and tomato transcriptome changes in response to *B*. *cinerea* infection. AbuQamar et al. [31] and Asselbergh et al. [32] studied the expression profiles of *B*. *cinerea*-inoculated plants to examine the defense transcriptome and identify genes involved in plant responses to the pathogen. Both studies found that abscisic acid plays an important role in plant resistance to *B*. *cinerea*. In our study, we also identified several genes involved in the abscisic acid pathway with significant changes in expression. Besides abscisic acid, many other plant hormones play important roles in resistance to *B*. *cinerea* [33, 34]. For example, ET and auxin are known to be involved in *Arabidopsis* defense against necrotrophic pathogens. Several cucumber genes involved in ET and auxin synthesis are also differentially expressed.

ROS are one of the most important plant defense responses to pathogens, although they lead to host cell death and facilitate necrotrophic colonization [35]. They can also cause cell wall modification, which is known to be an essential component of the plant defense response, and effectively block early development of *B*. *cinerea* [7, 31]. In our study, ROS formatting was observed in cucumber during *B*. *cinerea* attack. Nineteen cucumber DEGs were upregulated in the peroxisome pathway and may play an important role in cucumber defense against *B*. *cinerea*.

Recently, several studies have supported the hypothesis that fine regulation of antioxidant systems is part of the defense response signaling pathway [36]. The alpha-linolenic acid molecules contain three conjugated double bonds and thus showed very strong reducibility. In our analysis, 14 upregulated and 2 downregulated genes were involved in alpha-linolenic acid metabolism. We assumed that linolenic acid plays an important role in eliminating excessive accumulation of ROS. In addition, these 14 genes are candidate genes in regulating alpha-linolenic acid metabolism. However, most studies have explored GSH. Guo et al. [37] showed that depletion of GSH induces the accumulation of phytoalexins. De Gara et al. [36] observed a decrease in GSH content on tomato leaves infected with the necrotrophic *B*. *cinerea* and hypothesized that GSH plays an important role in resistance against *B*. *cinerea*. In our KEGG enrichment analysis, we also identified 27 DEGs involved in the GSH metabolism pathway. GSH is known to contain a mercapto group and may remove the peroxide produced by oxidative bursts and activate pathogen-related genes.

Photosynthesis is an essential process for plant growth. After inoculation with *B*. *cinerea*, the most enriched downregulated pathway was photosynthesis. Other associated pathways that were downregulated include “photosynthesis—antenna proteins” and “porphyrin and chlorophyll metabolism.” Genes involved in “photosynthetic membrane”, “photosystem”, “photosystem II”, and “photosynthesis” were also downregulated. Bilgin et al. [38] compared transcriptome data from microarray experiments after 22 different forms of biotic damage on 8 different plant species. They found that after plant damage to foliage by biotic agents, genes involved in photosynthesis were downregulated. Our data are consistent with those of previous studies, confirming that photosynthesis plays an important role in pathogen resistance.

For cucumber resistance *B*. *cinerea*, there were several DEGs involved in “evasion or tolerance of host immune response”, “evasion or tolerance of defense response of other organism involved in symbiotic interaction”, “response to defenses of other organism involved in symbiotic interaction”, these DEGs may play an important role in plant denfense pathogen. Especially Csa2G010390 gene belongs to “defense response to fungus” term was the only gene. It was annotated as pathogenesis-related protein, indicating that Csa2G010390 was a candidate gene in cucumber defense *B*. *cinerea*.

There was also an important pathway for cucumber defense *B*. *cinerea*. 26 cucumber DEGs were found involved in plant-pathogen interaction pathway (S1 Fig). Among them 18 DEGs were upregulated, 8 DEGs were downregulated. Through the analysis of this pathway, hypersensitivity response (HR), cell wall reinforcement, stomatal closure and defense-related gene induction were found that to have had great relevance with the concentration of calcium ions. Csa2G035490 gene annotated as probable cyclic nucleotide-gated ion channelion channels 5 protein was the only upregulated DEG in “CNGCs” node. Upregulation of this gene would promote the increasement of the calcium ion concentration, indicating that this gene had played an important role in cucumber defense *B*. *cinerea*.

During a pathogen attack, the plant should save energy to synthesize specific defense proteins. Cell walls are not only barriers that can prevent the proliferation of pathogens, but are also an important reservoir of energy-rich sugars [39]. Glucose metabolism is considered to be the basic characteristic of *B*. *cinerea* to infect plant. Thus, for pathogens to invade plant cells or exploit the polysaccharides of plant cell walls, they must secrete enzymes to disassemble cell wall polysaccharides. GO enrichment analysis showed that several glycosylation-related GO terms were significantly changed, including “hydrolase activity, hydrolyzing O-glycosyl compounds”, “hydrolase activity, acting on glycosyl bonds”, “cellulose binding”, and “polysaccharide binding.” Genes corresponding to these terms may play an important role in destroying the structure of plant cell walls. In addition, Blanco-Ulate et al. [39] used RNAseq to investigate which *B*. *cinerea* carbohydrate-active enzymes were expressed during infection of lettuce leaves, ripe tomato fruit, and grape berries. They found that *BcPG1*, *BC1G_14702*, *BcPME2*, *BcCel5A*, *BC1G_13862*, and *BC1G_12859* were highly expressed in these three hosts. The first five genes were consistent with our results, but *BC1G_12859* did not show differential expression in our study. It is possible that the function of the *BC1G_12859* gene is not crucial. In addition, there were several DEGs involved in “Galactose metabolism”, “Pentose and glucuronate interconversions”, “Fructose and mannose metabolism” pathways, indicating that these DEGs were *B*. *cinerea* virulence factors.

Excluding secreting cell wall—degrading enzymes, *B*. *cinerea* also produces toxic metabolites that induce cell death in advance of the invading hyphae [40]. These toxins are all secondary metabolites of *B*. *cinerea*. In our KEGG analysis, there were 102 genes involved in the “biosynthesis of secondary metabolites” pathway. In GO enrichment analysis, we also identified several genes associated with the production of toxins, including “toxin biosynthetic processes”, “mycotoxin metabolic processes”, “aflatoxin biosynthetic processes”, and “toxin metabolic processes.” It is possible that these DEGs, which are involved in the KEGG pathway and GO terms, were the candidate genes. Further studies are required to determine the function of these genes.

In addition, there were some new discoveries. B0510_6905, annotated as a hypothetical protein (BC1G_15833), was the only DEG involved in “evasion or tolerance of host defense response” term, indicating that B0510_6905 was a crucial gene in *B*. *cinerea* attacking cucumber. There were 12 upregulated and 7 down regulated DEGs involved in “pathogenesis”, indicating that all these 19 DEGs had a high revelance with *B*. *cinerea*’s virulence.

The *B*. *cinerea* whole-genome information is available, and we obtained the results of gene annotation by aligning the reads onto the reference genome. The functions of many of the proteins encoded by DEGs are unclear. Proteomics should be combined with our data to verify the function of these proteins.

In addition, through GO term analysis and KEGG pathway analysis, several DEGs involved in plant-pathogen intraction related GO terms and KEGG pathways had been indicated that play important roles in cucumber-*B*.*cinerea* interaction. Further study should be conducted to further validate the function of these genes, such as gene knockout technology.

With the development of biotechnology, our understanding of the mechanisms of the plant—pathogen interaction is increasing. In addition, we can examine the mechanisms of plant resistance to pathogens and mechanisms of pathogen attack on plants. Several studies have explored the co-evolution of plants and pathogens and have shown that the coevolution of plants and microbes shape plant mechanisms that detect and repel pathogens [41]. In addition, plants are engaged in a continuous co-evolutionary struggle for dominance with their pathogens [42]. Zhu et al. [43] assessed a computational framework based on a mixture model-based likelihood equipped with functionality to cluster genes based on dynamic and functional changes of gene expression within an interconnected system composed of the plant and pathogen. This allowed them to obtain a quantitative understanding of the plant—pathogen interaction at the transcriptional level. This framework could also be applied to our data to study plant—pathogen interactions.

## Supporting Information

S1 Fig Cucumber DEGs involved in the plant-pathgen interaction pathway. Red indicates significantly increased expression in cucumber innoculated with *B*. *cinerea* compared with controled cucumber; green indicates significantly decreased expression; yellow indicates both up-and down-regulated genes.
(TIF)

S1 Table Primers designed for RT-PCR. (DOCX)

S2 Table Details of differentially expressed genes of cucumber. (XLS)

S3 Table Details of differentially expressed genes of *B*. *cinerea*. (XLS)

S4 Table GO enrichment results of cucumber DEGs. (XLS)

S5 Table GO enrichment results of *B*. *cinerea* DEGs. (XLS)

S6 Table KEGG enrichment results of cucumber DEGs. (XLS)

S7 Table KEGG enrichment results of *B*. *cinerea* DEGs. (XLS)
