# Supplementary figures and images for: Large-Scale Transcriptome Analysis of Cucumber and Botrytis cinerea during Infection
Source: PLoS One. 2015 Nov 4;10(11):e0142221. doi: 10.1371/journal.pone.0142221 (PMC4633151; doi:10.1371/journal.pone.0142221)

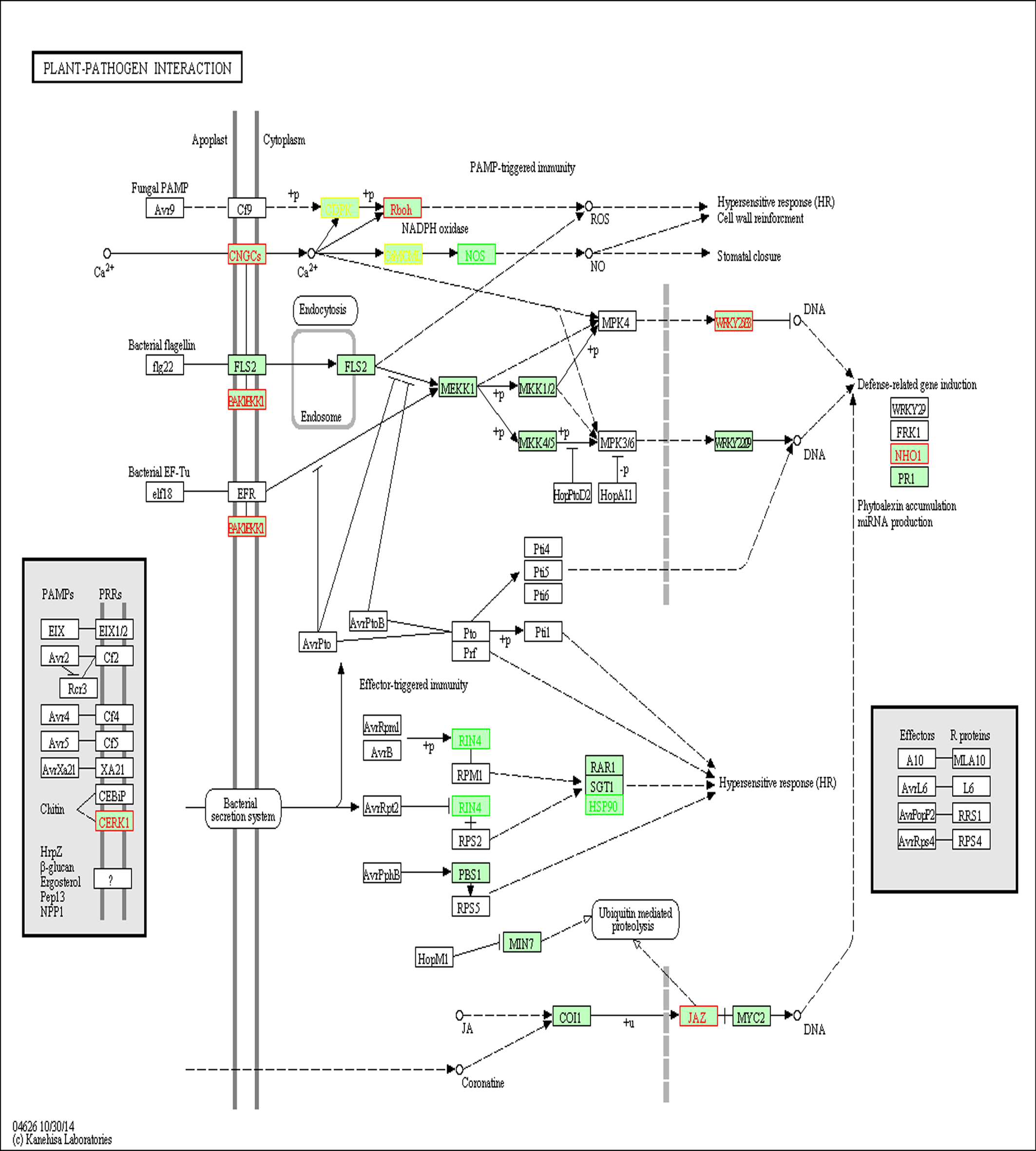

Supplement: S1 Fig — Red indicates significantly increased expression in cucumber innoculated with B. cinerea compared with controled cucumber; green indicates significantly decreased expression; yellow indicates both up-and down-regulated genes. (TIF) [file pone.0142221.s001.tif]
