# Supplementary material for: Large-Scale Transcriptome Analysis of Cucumber and Botrytis cinerea during Infection
Source: PLoS One. 2015 Nov 4;10(11):e0142221. doi: 10.1371/journal.pone.0142221 (PMC4633151; doi:10.1371/journal.pone.0142221)
Supplement: S1 Table — (DOCX) [file pone.0142221.s002.docx]

| **Gene ID** | **Forward sequence** | **Reverse sequence** |
| --- | --- | --- |
| Csa5G165850 | TGATGCTCGCCGCCGTTCTA | GCCGTTGTCGCACTCCTCTAT |
| Csa3G198490 | TTCTCCAACGCAACCAAGTCCT | AGAGGTAATCCGCCGCTGTAGA |
| Csa1G600830 | GCCAGTTTCTCACTTGCCAGAG | GCCTCCGCTCCATAGTCTTCTC |
| Csa1G008570 | TGCGATGCGAGACCAAGACAA | GCCATAAGCCGACATAGCCTTC |
| Csa1G534750 | CGGGAACGGCGAGGATTTCT | TGAGCAAAGAAGGCAGCGATTT |
| actin3 | GGCAGTGGTGGTGAACATG | TTCTGGTGATGGTGTGAGTC |
| B0510_2072 | GAACCACTGCCGATTCCGACTT | TCACCAGACGCCGAGTTAGGAG |
| B0510_8612 | CTGCCGCCGCTAGTACGAAT | CGCCATTAAGTGCCAGGAATCA |
| B0510_8082 | CCCAAGTCCGTGGTCCAAGAA | ATCCGATGAGTAGAGGCGAAGC |
| B0510_958 | ACAACACTCGCCACAAGGATGT | CACCGACCAAAGCAGCACCAA |
| B0510_903 | CCTGTAGCAACCGAGCAAGAGT | GCCCAGATAGCGAAATGTGACC |
| actin | AGCGTGAAATCGTCCGTGAT | GACTGGCGGTTTGGATTTCTT |

**Table S1 Primers designed for RT-PCR**
